# Association of Calcium and Phosphate Levels with Incident Chronic Kidney Disease in Patients with Hypoparathyroidism: A Retrospective Case-Control Study

**DOI:** 10.1155/2022/6078881

**Published:** 2022-11-02

**Authors:** Elvira O. Gosmanova, Olulade Ayodele, Kristina Chen, Erin E. Cook, Fan Mu, Joshua A. Young, Lars Rejnmark

**Affiliations:** ^1^Albany Medical College, Albany, NY, USA; ^2^Takeda Pharmaceuticals USA, Inc., Lexington, MA, USA; ^3^Analysis Group, Inc., Boston, MA, USA; ^4^Aarhus University and Aarhus University Hospital, Aarhus, Denmark

## Abstract

**Objective:**

Reasons for the increased incidence of chronic kidney disease (CKD) in patients with chronic hypoparathyroidism are poorly understood. This study evaluated associations between levels of albumin-corrected serum calcium, serum phosphate, and calcium-phosphate product and the odds of CKD development in patients with chronic hypoparathyroidism.

**Design:**

A retrospective nested case-control study of adult patients with chronic hypoparathyroidism who had ≥1 prescription for calcitriol who developed CKD and matched controls who did not develop CKD were selected from the IBM® Explorys electronic medical record database. *Patients*. The study included a cohort of 300 patients for the albumin-corrected serum calcium analysis and 80 patients for the serum phosphate and calcium-phosphate product analyses. *Measurements*. We examined associations between albumin-corrected serum calcium, serum phosphate and calcium-phosphate product levels, and the risk of devloping CKD (defined as ≥2 outpatient estimated glomerular filtration values <60 mL/min/1.73 m^2^ occuring ≥3 months apart or ≥1 diagnostic code for CKD stages 3–5).

**Results:**

Individuals who had ≥67% of albumin-corrected serum calcium measurements outside, above, or below the study-defined range (2.00–2.25 mmol/L [8.0–9.0 mg/dL]) had 3.5-, 2.9-, and 2.7-fold higher odds of developing CKD (adjusted odds ratios [95% CI]: 3.46 [1.82–6.56], 2.85 [1.30–6.28], and 2.68 [1.16–6.15]), respectively, compared with patients who had <33% of albumin-corrected calcium measurements in those ranges. There was no association between developing CKD and having any serum phosphate measurements or any calcium-phosphate product measurements above normal population ranges.

**Conclusion:**

In adult patients with chronic hypoparathyroidism, a higher proportion of albumin-corrected calcium measurements outside of the 2.00–2.25 mmol/L (8.0–9.0 mg/dL) range was associated with higher odds of developing CKD.

## 1. Introduction

Chronic hypoparathyroidism is a rare disease caused by absent or insufficient production of parathyroid hormone (PTH) [1]. PTH is an important regulator of serum calcium and phosphate concentrations [2]. Serum calcium levels are tightly controlled through a negative feedback mechanism; when calcium levels fall, calcium-sensing receptors in the parathyroid glands signal the release of PTH [3, 4]. In the kidney, PTH stimulates reabsorption of calcium, facilitates phosphate excretion, and enhances the conversion of 25-hydroxyvitamin D to 1,25-dihydroxyvitamin D [2, 3, 5]. The presence of 1,25-dihydroxyvitamin D contributes to the absorption of calcium and phosphate in the gastrointestinal tract [2, 5]. In addition to the recognized loss of the phosphaturic effect of PTH, the calcium-conserving PTH effects on the renal tubule are lost [1, 6]. In the bone, PTH stimulates the release of calcium and phosphate; the release of calcium from a labile skeletal pool can maintain serum calcium levels [7]. When PTH levels are inappropriately low or absent, hypocalcemia and hyperphosphatemia frequently ensue [1, 8].

Current guidelines provide a framework for biochemical parameter management in patients with chronic hypoparathyroidism that include maintenance of (i) serum calcium in a target range within the lower part of the normal population reference range of 2.15–2.55 mmol/L (8.6–10.2 mg/dL), while avoiding signs and symptoms of hypocalcemia, to minimize hypercalciuria and potential renal damage; (ii) serum phosphate levels within the normal population reference range of 0.81–1.45 mmol/L (2.5–4.5 mg/dL) to avoid hyperphosphatemia, which is a disease characteristic attributed to the lack of the phosphaturic PTH effect; (iii) a calcium-phosphate product <4.40 mmol^2^/L^2^ (<55 mg^2^/dL^2^) [1, 9, 10]. Guidelines also recognize that hyperphosphatemia may result in the deposition of calcium-phosphate complexes even when calcium-phosphate product does not exceed 4.40 mmol^2^/L^2^ (55 mg^2^/dL^2^) [1]. Conventional therapy for chronic hypoparathyroidism consists of oral calcium supplements and active vitamin D, which can correct hypocalcemia but does not replace other physiologic functions of PTH [1, 3]. In some patients, conventional therapy fails to adequately control hypoparathyroidism. Patients remain symptomatic, with episodes of hypocalcemia and hypercalcemia, and may develop complications, including chronic kidney disease (CKD) [1, 3, 11–14].

Assessment of kidney function using the estimated glomerular filtration rate (eGFR) is important in establishing a diagnosis of CKD [15]. In the absence of hormone replacement therapy, the increase in calcium influx resulting from oral calcium and active vitamin D supplementation is not counterbalanced by the calcium-conserving and phosphaturic effects of PTH. This can lead to transient periods of hypercalcemia and hypercalciuria [1, 3]. An increasing body of evidence indicates that patients with chronic hypoparathyroidism treated with conventional therapy are at increased risks for nephrolithiasis, nephrocalcinosis, developing a decline in eGFR, and progression to end-stage kidney disease [11, 14, 16–23]. However, despite evidence for an association between CKD and hypoparathyroidism, little is known about which factors mediate this association. We hypothesized that a frequent variation in serum calcium and phosphate, as assessed through longitudinal evaluation of biochemical parameters (i.e., calcium, phosphate, and calcium-phosphate product), may be associated with a higher risk of developing CKD. This retrospective study evaluated the association between levels of albumin-corrected serum calcium, serum phosphate, and calcium-phosphate product with odds of developing CKD in adult patients with chronic hypoparathyroidism.

## 2. Methods

### 2.1. Study Design

**Figure 1:**
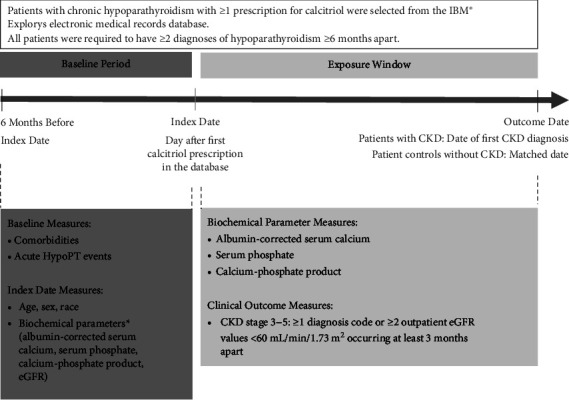
Study Design. CKD: chronic kidney disease; eGFR: estimated glomerular filtration rate; HypoPT: hypoparathyroidism.  ^*∗*^Value closest to the index date.

This was a retrospective, risk-set sampled, nested case-control study that assessed the association of biochemical parameters with odds of developing CKD in adult patients with chronic hypoparathyroidism in the United States (Figure 1). The risk-set sampling process allowed for patients at risk of developing CKD to be eligible for selection as a potential control during the time window before a diagnosis of CKD occurred and allowed the patients to be matched more precisely for exposure window duration. Patients with chronic hypoparathyroidism were identified from the IBM® Explorys electronic medical record database (January 2007 to August 2019). Explorys is a nationally representative database that covers about 15% of the US population. At the time of this analysis, data were obtained from approximately 360 different hospitals and 330,000 different healthcare providers and physicians. The deidentified data in Explorys represent ambulatory, inpatient, and postacute settings and also included laboratory measurements, diagnoses, procedures codes, medications, and demographic data. Because deidentified data from Explorys are compliant with the Health Insurance Portability and Accountability Act (HIPAA), ethics committee approval and informed consent were not required.

All patients selected from the database were required to have ≥2 diagnosis codes for hypoparathyroidism occurring ≥6 months apart to ensure that all patients had chronic and not transient hypoparathyroidism. Hypoparathyroidism was identified in the medical claims using International Classification of Diseases-Clinical Modification (ICD-CM) codes: ICD-9-CM code 252.1 and ICD-10-CM codes E20.0, E20.8, E20.9, and E89.2. All patients were required to have ≥1 prescription for calcitriol; the day after the first calcitriol prescription was defined as the index date. At index, patients were required to be ≥ 18 years old with data for a baseline period extending ≥6 months before the index date. Exclusion criteria at the index date were recombinant human parathyroid hormone (1–84), rhPTH (1–84) or teriparatide treatments at any time; or parathyroid cancer, esophageal cancer, lymphoma, or record of CKD (defined by ≥ 1 diagnosis code for CKD or ≥1 eGFR value <60 mL/min/1.73 m^2^) before the index date. The eGFR was estimated using the Chronic Kidney Disease-Epidemiology Collaboration (CKD-EPI) equation (GFR = 141 × min[Scr/*κ*, 1]^*α*^ × max[Scr/*κ*, 1]^−1.209^ × 0.993^age^ × 1.018 [if female] × 1.159 [if Black] where Scr is serum creatinine in mg/dL; *κ* is 0.7 for females and 0.9 for males; *α* is −0.329 for females and −0.411 for males; min indicates the minimum of Scr/*κ* or 1, and max indicates the maximum of Scr/*κ* or 1) [24].

After the index date, the censoring date for CKD was the first diagnosis of CKD, defined as the first eGFR value <60 mL/min/1.73 m^2^ or first diagnosis code. The exposure window was the time between the index date and first diagnosis of CKD. The outcome date was the date of first indication of development of CKD after the index date or a matched date for controls without CKD. The CKD outcome was defined as ≥2 outpatient eGFR values of <60 mL/min/1.73 m^2^ occurring ≥3 months apart or ≥1 diagnosis ICD-CM code for CKD stages 3–5. ICD-9 codes for CKD included the following: 585.1, 585.2, 585.3, 585.4, 403.01, 403.11, 403.91, 404.02, 404.03, 404.12, 404.13, 404.92, 404.93, 585.5, 585.6, 403.00, 403.10, 403.90, 404.00, 404.01, 404.10, 404.11, 404.90, 404.91, and 585.9. ICD-10 codes for CKD included the following: N18.1, N18.2, N18.3, N18.4, N18.5, N18.6, I13.11, I13.2, N18.9, I12.9, I13.0, and I13.10.

### 2.2. Cohorts and Statistical Analyses

Patients were those with CKD after the index date, and matched controls were those without CKD. Matching was 1:1 based on age ± 5 years, sex, index date ± 1 year, and exposure window duration. Separate patient cohorts were generated for the albumin-corrected serum calcium analysis and for the serum phosphate and calcium-phosphate analyses because of varying availability of laboratory measurements and sample size. For the albumin-corrected serum calcium sample, patients were required to have ≥2 albumin and serum calcium measurements occurring on the same day in the exposure window. For the phosphate sample, patients were required to have ≥1 set of calcium and phosphate measurements occurring on the same day during the exposure window and ≥1 phosphate measurement during the baseline period. No formal power calculation was performed; all patients who met the inclusion criteria and could be matched were included in the analyses.

Patient demographics, clinical characteristics, and biochemical parameters were compared between patients with CKD and controls using Wilcoxon signed-rank tests for continuous variables and McNemar tests for categorical variables; a threshold for statistical significance of *P* < 0.05 was applied for all tests. Continuous variables were reported as means with standard deviation (SD), with categorical variables as frequencies and proportions. Levels of albumin-corrected serum calcium, serum phosphate, and calcium-phosphate product during the exposure window were compared between patients with CKD and controls. The ranges used in this study were based on treatment guidelines for patients with chronic hypoparathyroidism [1, 9, 10]. The target range for albumin-corrected serum calcium in patients with chronic hypoparathyroidism used in this study was defined as 2.00–2.25 mmol/L (8.0–9.0 mg/dL), which is different from the normal population reference range (2.15–2.55 mmol/L; 8.6–10.2 mg/dL) [9]. In contrast, the reference ranges in patients with chronic hypoparathyroidism for serum phosphate (0.81–1.45 mmol/L; 2.5–4.5 mg/dL) and for calcium-phosphate product (≤4.40 mmol^2^/L^2^; ≤55 mg^2^/dL^2^) do not differ from normal population reference ranges. For the albumin-corrected serum calcium cohort, the proportions of measurements outside (above or below), above, and below the range were reported. For the serum phosphate cohort and the calcium-phosphate product cohort, the proportion of patients with measurements above the upper level of the normal range was reported. For each parameter, the time-weighted mean level was also described. Separate multivariable conditional logistic regression models were used to examine the association between albumin-corrected serum calcium, serum phosphate, and calcium-phosphate product and development of CKD. The albumin-corrected serum calcium models were adjusted for the following baseline characteristics: age, index year, race, hypertension, type 2 diabetes, any acute hypoparathyroidism events, and the Charlson Comorbidity Index (CCI). Acute hypoparathyroidism events were defined as any event of cardiac dysrhythmia, hypercalcemia, hypocalcemia, laryngeal spasm, muscle spasm, other convulsions, palpitations, tachycardia, tetanic cataract, or tetany using ICD-9-CM and ICD-10-CM codes. Sensitivity analyses examining the time-weighted mean level of albumin-corrected serum calcium were conducted with the same covariate adjustments as those used in the main analysis. The time-weighted mean level of albumin-corrected serum calcium was divided into tertiles of ≤2.07, 2.08–2.19, and ≥2.20 mmol/L. Among the patients in the albumin-corrected serum calcium cohort, the regression model was repeated in another sensitivity analysis among a subgroup restricted to patients with ≥1 eGFR measurement before the index date and measurements after the index date.

The serum phosphate and calcium-phosphate product models were adjusted for the following baseline characteristics: age, index year, baseline serum phosphate, hypocalcemia, and type 2 diabetes. Sensitivity analyses examining the time-weighted mean level of serum phosphate or calcium-phosphate product levels were conducted using the same covariates as those used in the main analyses. The time-weighted mean serum phosphate level was divided into tertiles of ≤1.31, 1.32–1.47, and ≥1.48 mmol/L, and time-weighted mean calcium-phosphate product levels were divided into tertiles of ≤2.75, 2.76–3.15, and ≥3.16 mmol^2^/L^2^. All statistical analyses were carried out using R version 3.6.2 software (The R Foundation, Vienna, Austria).

## 3. Results

**Figure 2:**
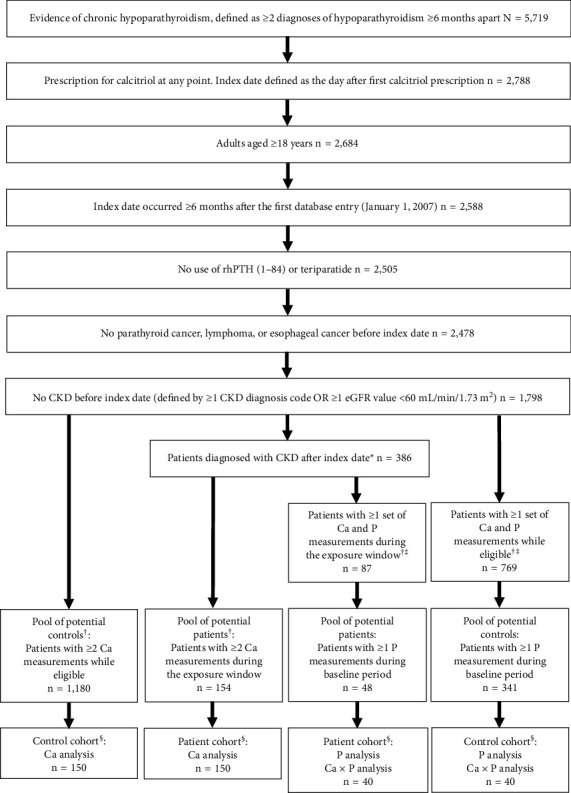
Selection of the study analysis cohorts. Ca: albumin-corrected serum calcium; Ca × P: calcium-phosphate product; CKD: chronic kidney disease; eGFR: estimated glomerular filtration rate; P: serum phosphate; rhPTH(1–84): recombinant human parathyroid hormone (1–84).  ^*∗*^Patients with CKD must have had a diagnosis of CKD after the index date to be eligible. Patients with a diagnosis of CKD before the index date were excluded. The first indicator of CKD after the index date was defined as the outcome date for patients who developed CKD, and a matched date was chosen for controls. ^†^The exposure window for patients who developed CKD was the time from the index date to the first diagnosis of CKD (the first eGFR <60 mL/min/1.73 m^2^ or the first CKD diagnosis code). The eligible window for patient controls was the time from the index date to the end of data availability or the day before CKD diagnosis, whichever occurred earlier. In risk-set sampling, patients who developed CKD were eligible to be chosen as potential controls during the time window before CKD diagnosis. ^‡^Patients with CKD and controls analyzed for the exposures of P and Ca × P were those who had at least one set of measurements for Ca and P available on the same day. ^§^Matched 1:1 on age ± 5years, sex, index date ± 1 year, and duration of exposure window.

The study included a cohort of 300 patients for the serum calcium analysis and a cohort of 80 patients for the serum phosphate or calcium-phosphate product analyses (Figure 2). Overall, patients with CKD and controls without CKD had similar baseline characteristics (Table 1). In the albumin-corrected serum calcium cohort, patients with CKD and controls had a similar mean age: mean ± SD age of 57.7 ± 12.8 versus 57.8 ± 12.8 years, respectively (*P*=0.78). In the serum phosphate and calcium-phosphate cohorts, patients with CKD and controls also had a similar mean age: mean ± SD age of 57.2 ± 12.4 and 57.6 ± 12.8 years, respectively (*P*=0.28). The mean exposure window duration was 2.8 years for the serum calcium analysis and 2.5 years for the serum phosphate and calcium-phosphate product analyses. During the exposure window, in the albumin-corrected serum calcium cohort and the phosphate and calcium-phosphate cohort, the first documented CKD diagnosis in patients was at stage 3 in 97.3% and 92.5% of patients, stage 4 in 2.0% and 7.5%, and stage 5 in 0.7% and 0%, respectively.

### 3.1. Albumin-Corrected Serum Calcium, Serum Phosphate, and Calcium-Phosphate Product Levels

During the exposure window, patients with CKD compared with controls without CKD had a similar mean number of measurements of albumin-corrected serum calcium (7.3 vs 7.0, *P*=0.61), serum phosphate (6.0 vs 5.9, *P*=0.94), and calcium-phosphate product (4.8 vs 6.3, *P*=0.72). Patients with CKD compared with controls had a higher proportion of albumin-corrected serum calcium measurements outside, above, or below the 2.00–2.25 mmol/L (8.0–9.0 mg/dL) range (57.9% vs 41.2%, *P* < 0.001; 30.1% vs 20.0%, *P* < 0.01; 27.8% vs 20.1%, *P*=0.09, respectively; Supplementary Figure 1A). There were no differences in the proportion of patients with CKD compared with controls who had any serum phosphate measurements above the 0.81–1.45 mmol/L (2.5–4.5 mg/dL) range (65% vs 62.5%, *P*=1.00) or any calcium-phosphate product measurements above 4.40 mmol^2^/L^2^ (55 mg^2^/dL^2^; 12.5% vs 10.0%, *P*=1.00; Supplementary Figure 1B).

### 3.2. Multivariable Regression Model Analyses

#### 3.2.1. Albumin-Corrected Serum Calcium

In analyses including matched groups (Table 2), patients with ≥67% of albumin-corrected serum calcium measurements outside, above, or below 2.00–2.25 mmol/L (8.0–9.0 mg/dL) had higher odds of developing CKD compared with patients with <33% of albumin-corrected serum calcium measurements. In analyses adjusted for demographic characteristics and comorbidities (Table 2), patients with ≥67% of albumin-corrected serum calcium measurements outside the 2.00–2.25 mmol/L (8.0–9.0 mg/dL) range had 3.5 times higher odds of developing CKD (adjusted OR, 3.46; 95% CI, 1.82–6.56; *P* < 0.001) compared with patients with <33% of albumin-corrected serum calcium measurements outside this range. In addition, patients with ≥67% of measurements above this range had 2.9 times higher odds of developing CKD (adjusted OR, 2.85; 95% CI, 1.30–6.28; *P* < 0.01) compared with patients with <33% of measurements above the range. Likewise, patients with ≥67% of measurements below this range had 2.7 times higher odds of developing CKD (adjusted, OR 2.68; 95% CI, 1.16–6.15; *P*=0.02) than patients with <33% of measurements below the range. Table 2 presents outcomes for patients who had 33% to <67% of their albumin-corrected serum calcium measurements outside, above, or below 2.00–2.25 mmol/L (8.0–9.0 mg/dL) compared with patients who had <33% of measurements in the corresponding categories. There was a trend for increased risk of CKD in the former groups, but it did not reach statistical significance.

#### 3.2.2. Serum Phosphate and Calcium-Phosphate Product

In analyses including matched groups, there were no associations between the development of CKD and having any serum phosphate or any calcium-phosphate measurements above the range (Table 3). In adjusted regression analyses (Table 3), there was also no association between the development of CKD and having any serum phosphate measurements above 0.81–1.45 mmol/L (2.5–4.5 mg/dL) or any calcium-phosphate product measurements above 4.40 mmol^2^/L^2^ (55 mg^2^/dL^2^) in patients with CKD compared with controls.

### 3.3. Sensitivity Analyses

Two sensitivity analyses were performed. In the first sensitivity analysis that examined time-weighted mean levels (Table 4), there were no associations between tertiles of albumin-corrected serum calcium, phosphate, or calcium-phosphate product and the development of CKD.

Another sensitivity analysis was performed among patients with eGFR available at the baseline and during the exposure window and included 89 patients with CKD and 89 matched controls without CKD. Patients were of similar age (55.6 ± 11.9 vs 56.0 ± 12.2 years; *P* = 0.27) and had a similar CCI score (1.6 ± 2.5 vs 1.9 ± 2.3; *P* = 0.34); however, they had a higher proportion of albumin-corrected serum calcium measurements outside the 2.00–2.25 mmol/L (8.0–9.0 mg/dL) range compared with controls (61.1% vs 45.5%; *P* < 0.01). Patients with ≥67% of albumin-corrected serum calcium measurements outside or above the 2.00–2.25 mmol/L (8.0–9.0 mg/dL) range had 2.5- or 2.9-fold higher odds of developing CKD, respectively (outside: adjusted OR, 2.50; 95% CI, 1.15–5.45; *P* = 0.02; above: adjusted OR, 2.85; 95% CI, 1.03–7.89; *P* = 0.04), compared with patients with <33% of respective albumin-corrected serum calcium measurements. There was also 1.4-fold higher odds of developing CKD with ≥67% of albumin-corrected serum calcium measurements below the range (adjusted OR, 1.38; 95% CI, 0.62–3.07; *P* = 0.43), but this was not statistically significant compared to controls with <33% of respective albumin-corrected serum calcium measurements, possibly due to the reduced cohort size in the sensitivity analysis.

## 4. Discussion

Results of this study showed that there was a higher risk of the development of CKD among adult patients with chronic hypoparathyroidism who had a higher proportion of albumin-corrected serum calcium measurements outside (regardless if above or below) the 2.00–2.25 mmol/L (8.0–9.0 mg/dL) range. The sensitivity analysis that was restricted to individuals with an available baseline and follow-up eGFR also indicated these associations. These results were consistent with a Danish case-control study of 431 patients with hypoparathyroidism, identified from the Danish National Patient Registry, that found that patients with ≥4 hypercalcemic episodes had higher odds of renal disease (odds ratio, 3.3; 95% CI, 1.55–7.08) [11]. Both the current study and the Danish study found no association between time-weighted mean albumin-corrected serum calcium levels and development of CKD. The lack of association between time-averaged mean albumin-corrected serum calcium levels and risk of CKD could be related to the fact that mean levels may not accurately describe variability in calcium levels.

The current study did not find associations between serum phosphate measurements above the reference range (0.81–1.45 mmol/L [2.5–4.5 mg/dL]) or a higher time-weighted phosphate mean level with the odds of developing CKD. This serum phosphate analysis finding is also consistent with the Danish study, which found that the time-weighted average level of serum phosphate was not associated with higher odds of developing renal disease [11]. Elevated calcium-phosphate product has previously been found to be associated with cardiovascular disease [25]. The current study did not find that having any calcium-phosphate product measurement above 4.40 mmol^2^/L^2^ (55 mg^2^/dL^2^) was associated with development of CKD. In contrast, the Danish study found that a higher time-weighted average level of calcium-phosphate product was associated with higher odds of developing renal disease [11]. There were several differences between the current study and the Danish study. In the Danish study, ionized serum calcium was assessed and renal diseases were defined by diagnostic codes [11], whereas in the current study, albumin-corrected serum calcium was measured and CKD was identified by diagnosis codes or eGFR. In addition, the small cohort size of the phosphate and calcium-phosphate product cohort may have limited the ability to detect associations between higher levels of these parameters and development of CKD. Although the second and third tertiles of time-weighted serum phosphate and calcium-phosphate product were each associated with increasingly higher odds of developing CKD, these associations did not reach statistical significance.

Decline in eGFR is associated with a higher risk of developing end-stage renal disease [26]. In single-arm clinical trials in patients with chronic hypoparathyroidism treated 5 to 8 years with rhPTH(1–84), eGFR remained stable, albumin-corrected serum calcium was maintained within or slightly below the disease-management target range of 2.00–2.25 mmol/L (8.0–9.0 mg/dL) despite a significant reduction in the amount of supplemental oral calcium and active vitamin D, leading to decline in 24-hour urinary calcium from pretreatment levels, and serum phosphate was maintained within the normal population range (0.81–1.45 mmol/L; 2.5–4.5 mg/dL) [27, 28]. Moreover, a historical cohort of adult patients with chronic hypoparathyroidism derived from the United States-regional Geisinger Health System MedMining electronic health records database who were not treated with rhPTH(1–84) (*n* = 53) had significant decline in eGFR over a 5-year period compared to patients treated with rhPTH(1–84) in clinical trials (*n* = 69) [22]. In addition, patients receiving rhPTH(1–84) in REPLACE (a double-blind, placebo-controlled, randomized, phase 3 study) had a slight decrease in 24-hour urinary calcium excretion, while albumin-corrected serum calcium levels remained within the target range of 2.0–2.25 mmol/L (8.0–9.0 mg/dL) despite significant reductions in doses of oral calcium and active vitamin D compared with placebo [29]. Similarly, in the 5-year, open-label extension study, RACE, which evaluated rhPTH(1–84) in adults with chronic hypoparathyroidism, 24-hour urinary calcium decreased from the baseline, whereas albumin-corrected serum calcium remained within the target range [27]. It is plausible that the favorable effects of rhPTH(1–84) on kidney function may be due to the maintenance of calcium levels within the normal range and reduced calciuria. Therefore, replacement of missing PTH in chronic hypoparathyroidism, in order to maintain more stable serum calcium levels with lesser use of supplemental calcium, is an attractive option and requires further research.

The present study has both strengths and limitations. Strengths of the study include the detailed laboratory data for patients with chronic hypoparathyroidism, despite it being a rare condition. A nested case-control study design was used to match important confounders, including age, sex, and the exposure window duration. The study design also captured changes in the exposure variables over time, which allowed for assessment as a proportion of any biochemical parameters outside of the disease-relevant ranges. A robust statistical analysis was used to account for multiple potential baseline differences between patients who developed CKD and controls who did not develop CKD. However, given the retrospective design of this study, the presence of residual confounders cannot be excluded. In addition, the Explorys database only contained information about visits that occurred within the network of providers, so visits outside of the system were not captured. The scope of the analyses was limited by the information available in the database. eGFR data were not available for all patients and as such this may have resulted in misclassification of patients as not having CKD at the baseline or follow-up. However, the sensitivity analysis in which only patients with the baseline and follow-up eGFR were included showed similar results to the main analyses. Other laboratory values of interest, such as the urine albumin creatinine ratio, PTH concentration, and urinary calcium, were also not available for all patients in the study and were therefore not examined in this analysis. The monitoring of serum phosphate and calcium-phosphate product was infrequent in this study. This in turn limited the sample size and might have influenced the inability to uncover associations between serum phosphate and calcium-phosphate product and development of CKD. A potential limitation resulting from the use of albumin-corrected serum calcium is that the type of assay used to measure albumin is not standardized [30]. Therefore, albumin correction could have introduced variation in calcium levels that could influence the classification of values within or outside the target range. Evidence suggests that the measurement of ionized serum calcium provides a more accurate physiological assessment than albumin-corrected serum calcium [31]; however, there are technical issues associated with the measurement of ionized calcium that limit its availability, such as the need for a free-flowing source of venous blood, strict anaerobic collection conditions, immediate processing of the samples, and proper calibration of the measuring instrument [1, 30]. Importantly, current guidelines do not recommend ionized calcium over albumin-adjusted serum calcium for the monitoring of calcium levels [9]. Changes in oral calcium supplementation, adherence to conventional therapy regimens, and thiazide use were not evaluated because the data were not captured comprehensively in the database. In addition, due to possible over-the-counter use, calcium and calcitriol dosing in the data may not reflect the actual dosage taken by patients. The data in our analysis were left-censored, and no information on previous history of acute kidney injury or kidney stones, disease duration, severity, or etiology was available (i.e, surgical or nonsurgical); hence, stratified analyses could not be performed. However, patients with conditions associated with severe hypoparathyroidism, such as parathyroid cancer, esophageal cancer, and lymphoma, were excluded to produce a sample with a similar range of hypoparathyroidism etiologies.

In summary, in this nested case-control study of adult patients with chronic hypoparathyroidism investigating the association between biochemical parameters and development of CKD, patients who had a higher proportion of albumin-corrected serum calcium measurements outside/above/below of 2.00–2.25 mmol/L (8.0–9.0 mg/dL, i.e., disease-recommended lower than normal population reference range) had increased odds of developing CKD. In contrast, patients who had any serum phosphate measurements or any calcium-phosphate product measurements above normal population reference ranges did not have higher odds of developing CKD. Further studies are warranted to explore whether treatment modifications aiming to achieve more stable serum calcium levels within recommended disease management ranges may reduce the risk of CKD development in patients with chronic hypoparathyroidism.

## Figures and Tables

**Table 1:** Demographic, clinical characteristics, and biochemical parameter levels at the baseline in patients with chronic hypoparathyroidism and CKD and matched patient controls.

Parameter	Albumin-corrected serum calcium cohort (*n* = 300)			Phosphate and calcium-phosphate cohort (*n* = 80)		
	Patients with CKD (*n* = 150)	Control patients without CKD (*n* = 150)	*P* value	Patients with CKD (*n* = 40)	Control patients without CKD (*n* = 40)	*P* value
Demographics						
Age at index date ^*∗*^^†‡^ (years), mean ± SD	57.7 ± 12.8	57.8 ± 12.8	0.78	57.2 ± 12.4	57.6 ± 12.8	0.28
Female, *n* (%)	125 (83.3)	125 (83.3)	—	33 (82.5)	33 (82.5)	—
Race, ^†^*n* (%)						
White	125 (83.3)	129 (86.0)	0.61	33 (82.5)	32 (80.0)	1.00
Black	18 (12.0)	5 (3.3)	<0.01^¶^	6 (15.0)	4 (10.0)	0.68
Asian, multi, other, unknown	7 (4.7)	16 (10.7)	0.10	1 (2.5)	4 (10.0)	0.37
Average exposure window duration (years), mean ± SD	2.8 ± 2.2	2.8 ± 2.2	—	2.5 ± 2.3	2.5 ± 2.3	—
Clinical characteristics						
Comorbidities during baseline						
CCI score, ^†^mean ± SD	1.3 ± 2.3	1.6 ± 2.4	0.23	1.8 ± 3.0	1.7 ± 2.1	0.83
Type 2 diabetes, ^†‡^*n* (%)	20 (13.3)	16 (10.7)	0.56	2 (5.0)	5 (12.5)	0.45
Hypertension, ^†^*n* (%)	46 (30.7)	49 (32.7)	0.79	9 (22.5)	11 (27.5)	0.81
Any acute HypoPT events during baseline, ^†§^*n* (%)	60 (40.0)	56 (37.3)	0.71	21 (52.5)	21 (52.5)	1.00
Hypocalcemia, ^†‡^*n* (%)	43 (28.7)	38 (25.3)	0.58	16 (40)	19 (47.5)	0.66
Biochemical parameters, ^||^mean ± SD						
Albumin-corrected total serum calcium, mmol/L	2.0 ± 0.3	2.1 ± 0.3	0.82	1.9 ± 0.4	2.1 ± 0.3	0.19
Serum phosphate, ^‡^mmol/L	1.5 ± 0.4	1.5 ± 0.4	0.83	1.4 ± 0.4	1.4 ± 0.4	0.72
Calcium-phosphate product, mmol^2^/L^2^	2.8 ± 1.1	3.0 ± 0.9	0.46	2.9 ± 1.1	2.8 ± 0.8	0.70
eGFR, mL/min/1.73 m^2^	86 ± 14.0	91 ± 14.8	0.18	91 ± 12.4	87 ± 13.1	0.40

CCI: Charlson comorbidity index; CKD: chronic kidney disease; eGFR: estimated glomerular filtration rate; HypoPT: hypoparathyroidism.  ^*∗*^Index date was the day after the first calcitriol prescription. ^†^Albumin-corrected serum calcium regression models were adjusted for this parameter. ^‡^Serum phosphate and calcium-phosphate product regression models were adjusted for this parameter.^§^“Any acute HypoPT event” included hypercalcemia, hypocalcemia, laryngeal spasm, muscle spasm, other convulsions, tetanic cataract, tetany, cardiac dysrhythmias, palpitations, and tachycardia. ^||^Value closest to the index date. ^¶^Denotes *P* value < 0.05.

**Table 2:** Association between albumin-corrected serum calcium and development of CKD ^*∗*^.

	Patients with CKD (*n* = 150), *n*	Control patients without CKD (*n* = 150), *n*	Matched groups			Adjusted analyses		
			Odds Ratio	95% CI	*P* value	Odds Ratio	95% CI	*P* value
*Proportion of measurements outside of 2.00–2.25 mmol/L (8.0–9.0 mg/dL)* ^†^								
≥67%	63	27	3.39	1.81–6.38	<0.001^‡^	3.46	1.82–6.56	<0.001^‡^
33% to <67%	51	62	1.40	0.75–2.61	0.29	1.36	0.72–2.56	0.34
<33%	36	61	1.00	(Reference)	—	1.00	(Reference)	—

*Proportion of measurements above 2.00–2.25 mmol/L (8.0–9.0 mg/dL)* ^†^								
≥67%	25	11	2.61	1.21–5.59	0.01^‡^	2.85	1.30–6.28	<0.01^‡^
33% to <67%	37	31	1.47	0.85–2.53	0.17	1.53	0.87–2.70	0.14
<33%	88	108	1.00	(Reference)	—	1.00	(Reference)	—

*Proportion of measurements below 2.00*–*2.25 mmol/L (8.0–9.0 mg/dL)*^†^								
≥67%	26	10	2.75	1.21–6.25	0.02^‡^	2.68	1.16–6.15	0.02^‡^
33% to <67%	31	37	0.78	0.43–1.42	0.42	0.71	0.38–1.33	0.29
<33%	93	103	1.00	(Reference)	—	1.00	(Reference)	—

CCI: Charlson comorbidity index; CI: confidence interval; CKD: chronic kidney disease.  ^*∗*^Models were run for the proportion outside, above, and below the range. Matching for groups was 1:1 based on age ± 5 years, sex, index date ± 1 year, and exposure window duration. Adjusted models were adjusted for the following baseline characteristics: age (continuous variable), index year (continuous variable), race (White vs Black, Asian, multi, other, or unknown), hypertension, type 2 diabetes, any acute hypoparathyroidism event (event of cardiac dysrhythmia, hypercalcemia, hypocalcemia, laryngeal spasm, muscle spasm, other convulsions, palpitations, tachycardia, tetanic cataract, or tetany), and CCI. ^†^The target range for albumin-corrected serum calcium in patients with chronic hypoparathyroidism is defined as 2.00–2.25 mmol/L (8.0–9.0 mg/dL), which is different from the normal population reference range (2.15–2.55 mmol/L; 8.6–10.2 mg/dL). ^‡^Denotes any *P* value < 0.05.

**Table 3:** Association between serum phosphate and calcium-phosphate product and development of CKD ^*∗*^.

	Patients with CKD (*n* = 40), *n*	Control patients without CKD (*n* = 40), *n*	Matched groups			Adjusted analyses		
			Odds Ratio	95% CI	*P* value	Odds Ratio	95% CI	*P* value
*Any serum phosphate measurements above 0.81–1.45 mmol/L (2.5*–*4.5 mg/dL)*^*†*^								
Yes	26	25	0.97	0.34–2.73	0.95	0.95	0.32–2.86	0.93
No	14	15	1.00	(Reference)	—	1.00	(Reference)	—

*Any calcium-phosphate product measurements above 4.40 mmol* ^*2*^ * /L* ^*2*^ * (>55 mg* ^*2*^ * /dL* ^*2*^)^‡^								
Yes	5	4	1.07	0.26–4.39	0.93	0.79	0.16–4.01	0.78
No	35	36	1.00	(Reference)	—	1.00	(Reference)	—

CI: confidence interval; CKD: chronic kidney disease. Matching for groups was 1:1 based on age ± 5 years, sex, index date ± 1 year, and exposure window duration. Adjusted models were adjusted for the following baseline characteristics: age (continuous variable), index year (continuous), serum phosphate level in mmol/L (baseline measurement closest to index date, scaled 0.1-unit increase), hypocalcemia, and type 2 diabetes. ^†^The reference range for serum phosphate in patients with chronic hypoparathyroidism is 0.81–1.45 mmol/L (2.5–4.5 mg/dL), which does not differ from the normal population reference range. ^‡^The reference range for calcium-phosphate product in patients with chronic hypoparathyroidism is ≤ 4.40 mmol^2^/L^2^ (≤55 mg^2^/dL^2^), which does not differ from the normal population reference range.

**Table 4:** Sensitivity analysis multivariable regression models of time-weighted albumin-corrected serum calcium, serum phosphate, and calcium-phosphate product and development of CKD.

Time-weighted mean level		Patients with CKD, *n*	Control patients without CKD, *n*	Odds Ratio	95% CI	*P* value
*Tertiles of albumin-corrected serum calcium, * ^*∗*^^*†*^* (n* *=* *150 for patients and controls)*						
mmol/L	mg/dL					
≤2.07	≤8.28	50	50	1.11	0.62–1.97	0.73
2.08–2.19	8.32–8.76	46	54	1.00	(Reference)	—
≥2.20	≥8.80	54	46	1.47	0.82–2.66	0.20

*Tertiles of serum phosphate, * ^*‡§*^ * (n* *=* *40 for patients and controls)*						
mmol/L	mg/dL					
≤1.31	≤4.06	12	15	1.00	(Reference)	—
1.32–1.47	4.09–4.56	14	12	2.09	0.57–7.60	0.26
≥1.48	≥4.59	14	13	2.47	0.50–12.12	0.27

*Tertiles of calcium-phosphate product, * ^*‡||*^ * (n* *=* *40 for patients and controls)*						
mmol^2^/L^2^	mg^2^/dL^2^					
≤2.75	≤34.08	11	16	1.00	(Reference)	—
2.76–3.15	34.20–39.03	15	11	3.18	0.85–11.95	0.09
≥3.16	≥39.16	14	13	3.53	0.71–17.49	0.12

CCI: Charlson comorbidity index; CI: confidence interval; CKD: chronic kidney disease.  ^*∗*^Models were adjusted for the following baseline characteristics: age (continuous variable), index year (continuous variable), race (White vs Black, Asian, multi, other, or unknown), hypertension, type 2 diabetes, any acute hypoparathyroidism events, and CCI (continuous variable). ^†^The target range for albumin-corrected serum calcium in patients with chronic hypoparathyroidism is defined as 2.00–2.25 mmol/L (8.0–9.0 mg/dL), which is different from the normal population reference range (2.15–2.55 mmol/L; 8.6–10.2 mg/dL). ^‡^Models were adjusted for the following baseline characteristics: age (continuous variable), index year (continuous), serum phosphate level in mmol/L (baseline measurement closest to the index date, scaled 0.1-unit increase), hypocalcemia, and type 2 diabetes. ^§^The reference range for serum phosphate in patients with chronic hypoparathyroidism is 0.81–1.45 mmol/L (2.5–4.5 mg/dL), which does not differ from the normal population reference range. ^||^The reference range for calcium-phosphate product in patients with chronic hypoparathyroidism is ≤ 4.40 mmol^2^/L^2^ (≤55 mg^2^/dL^2^), which does not differ from the normal population reference range.

## Data Availability

The data used to support the study are included within the article.

## Supplementary Materials

Supplementary Materials Supplementary Figure 1: Proportion of Biochemical Parameter Measurements During the Exposure Window. Bar graph of unadjusted data for the (A) proportion of albumin-corrected serum calcium measurements outside, above, and below the range and (B) proportion of patients with any serum phosphate and any calcium-phosphate product measurements above the range. Albumin-corrected serum calcium: *n* = 150 each for patients and patient controls; serum phosphate and calcium phosphate: *n* = 40 each for patients and patient controls. Abbreviation: CKD, chronic kidney disease.
