# Supplementary figures and images for: Association of Calcium and Phosphate Levels with Incident Chronic Kidney Disease in Patients with Hypoparathyroidism: A Retrospective Case-Control Study
Source: Int J Endocrinol. 2022 Nov 2;2022:6078881. doi: 10.1155/2022/6078881 (PMC9646300; doi:10.1155/2022/6078881)

A

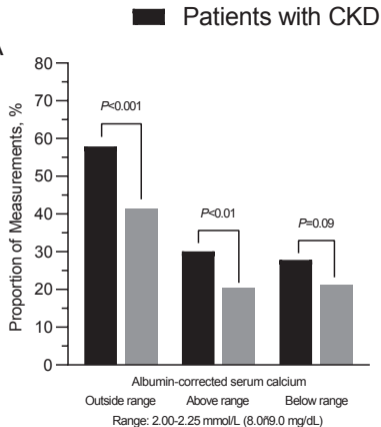

B

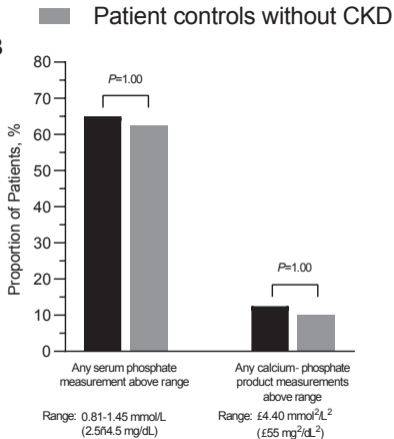

Supplement: Supplementary Materials — Supplementary Figure 1: Proportion of Biochemical Parameter Measurements During the Exposure Window. Bar graph of unadjusted data for the (A) proportion of albumin-corrected serum calcium measurements outside, above, and below the range and (B) proportion of patients with any serum phosphate and any calcium-phosphate product measurements above the range. Albumin-corrected serum calcium: n = 150 each for patients and patient controls; serum phosphate and calcium phosphate: n = 40 each for patients and patient controls. Abbreviation: CKD, chronic kidney disease. [file 6078881.f1.pdf]
